# Consensus-based guidelines for emotional intelligence interventions for first-year higher education students: a Delphi study

**DOI:** 10.3389/fpsyg.2026.1866661

**Published:** 2026-10-06

**Authors:** Fátima M. Teixeira, Alexandra M. Araújo, Rosario Cabello

**Affiliations:** 1 Department of Psychology and Education, CINTESIS.UPT@RISE-HEALTH, Portucalense University, Oporto, Portugal; 2 Department of Developmental and Educational Psychology, Faculty of Psychology, University of Málaga, Málaga, Spain

**Keywords:** Delphi study, emotional intelligence, first-year students, higher education, intervention guidelines

## Abstract

A successful transition to higher education (HE) is associated with cognitive, social, and emotional skills that help students navigate developmental, contextual, and academic challenges. Emotional Intelligence (EI) has been linked to psychological adjustment and academic functioning. However, few interventions targeting first-year students are guided by structured and theoretically coherent frameworks. This study aimed to develop consensus-derived guidelines for a tailored EI intervention designed to support emotional abilities, adaptation to HE, academic success, and well-being among first-year students. The proposed framework focuses on three core components: design, content, and efficacy assessment. Adopting a two-round modified e-Delphi method, a preliminary questionnaire was developed from an extensive review of systematic reviews, meta-analyses, and previously published interventions, and subsequently piloted by four specialists to ensure thematic relevance and clarity. An international panel of 12 experts in emotional competence and academic success in HE participated in iterative Delphi rounds to establish consensus regarding the intervention’s components and assessment procedures. Consensus was reached on a 10-session intervention delivered for 2 h once a week, incorporating team-based activities, role-plays, group discussions, and emotional literacy exercises, supported by tailored intervention materials and activities assigned between sessions. Regarding the intervention’s content, consensus was reached on integrating the four-branch EI abilities model throughout the intervention, alongside additional themes such as mental health and self-esteem. For assessment, experts also reached consensus on a mixed-methods approach, combining self-report and ability-based measures with focus groups and session-level assessments. A baseline assessment would be conducted 1 week before the intervention with at least one follow-up assessment. The findings highlight the experts’ perceived importance of structured socioemotional interventions tailored to the needs of first-year students in HE. This study provides preliminary, expert-informed guidelines that may support the future development and implementation of EI interventions in HE settings and contributes to ongoing discussions regarding the practical application of EI models in HE contexts, and underscores the need for future empirical validation and implementation research.

## 1 Introduction

Research has identified Higher Education (HE) students as a vulnerable population that faces an increased risk of developing mental health issues compared to their peers (Naylor et al., 2017; Sheldon et al., 2021). Recent studies indicate a prevalence between 25 to 50%, on average, of mental illnesses, such as depression and anxiety, among undergraduate students, a situation that has worsened after the COVID-19 pandemic (Amaro et al., 2024; Fruehwirth et al., 2021; Sheldon et al., 2021). Additionally, these students are at a higher risk of poor lifestyle habits, including insufficient sleep, lack of exercise, and unhealthy eating, along with a high incidence of substance use (Amaro et al., 2024; Sheldon et al., 2021). First-year students, in particular, often struggle with unfulfilled expectations, academic stress, feelings of loneliness, difficulties in establishing new meaningful relationships, and a weak connection to the university and their academic program, ultimately leading to academic failure and dropout (Araújo et al., 2019; Arnett et al., 2014; Naylor et al., 2017; Tinto, 2017; Tuero et al., 2024; Zanden et al., 2018).

To address these challenges, research on improving well-being and academic success among undergraduate students emphasizes the importance of emotional intelligence (EI) abilities, including emotional understanding and emotional regulation (Hashim et al., 2025; MacCann et al., 2020). These abilities are essential in providing young people with protective factors that help them navigate the stressful academic environment and personal challenges that come with enrolling in HE, thereby reducing the risk of mental health issues and promoting academic achievement (Hashim et al., 2025; MacCann et al., 2020). Undergraduate students who cultivate emotional abilities tend to achieve better grades, are more engaged in academic activities, and exhibit greater motivation toward their studies (MacCann et al., 2020). They also experience higher self-esteem, improved well-being, healthier behaviors, lower rates of depression, anxiety, and substance use, and better relations with peers and professors (MacCann et al., 2020; Sánchez-Álvarez et al., 2015; Schoeps et al., 2020; Tuero et al., 2024).

The most widely used frameworks for conceptualizing EI include the ability model and mixed models (Mayer and Caruso, 2025; Mayer et al., 2016). Recently, EI has also been classified according to the type of assessment used, distinguished between performance-based ability EI, self-report ability EI, and self-report mixed EI (Joseph and Newman, 2010; Mayer and Caruso, 2025). The four-branch model (Mayer and Salovey, 1997; Mayer et al., 2016; Salovey and Mayer, 1990) describes four branches of ability EI, each representing a distinct area of problem-solving. From the most cognitively basic to the most complex, these branches are: (a) perceiving emotions, (b) facilitating thought using emotions, (c) understanding emotions, and (d) managing emotions (Mayer et al., 2016). Regarding EI assessment, the performance-based ability model assesses EI by requiring participants to solve problems that have clear correct and incorrect answers (e.g., the Mayer–Salovey–Caruso Emotional Intelligence Test, MSCEIT; Mayer et al., 2003). In contrast, the self-report ability model assesses EI through self-assessment questionnaires, also based on the ability EI model (e.g., the Trait Meta-Mood Scale; Fernandez-Berrocal and Extremera, 2004; Salovey et al., 1995).

The present study adopts the four-branch ability model as its primary theoretical framework because it conceptualizes EI as a set of trainable competencies relevant to emotional adaptation and functioning in educational contexts with stable and lasting effects over time after training (Gong and Jiao, 2019; Hodzic et al., 2018; Kotsou et al., 2019; MacCann et al., 2020). In addition, we will also emphasize EI assessment tools based on this model, as research has indicated that ability EI assessed with such measures is associated with enhanced personal development, well-being, positive interpersonal relationships, and academic success (Llamas-Díaz et al., 2022; MacCann et al., 2020; Sánchez-Álvarez et al., 2015; Schoeps et al., 2020; Wong and Liem, 2022).

Given its malleability and potential for development through targeted educational experiences, EI has increasingly been considered a relevant target for preventive and developmental interventions in HE (Asiedu et al., 2025; Geßler et al., 2021; Zhoc et al., 2020). In this context, EI interventions have shown promising effects on students’ academic and psychological functioning, including academic engagement and well-being (Acebes-Sánchez et al., 2025; MacCann et al., 2020; Schoeps et al., 2020; Wong and Liem, 2022). Evidence from EI interventions in HE also suggests potential benefits for students’ emotional and mental health, including reduced perceived stress, anxiety, and depressive symptoms, as well as improved psychological well-being and the use of adaptive emotion regulation strategies (Acebes-Sánchez et al., 2025; Asiedu et al., 2025; Baik et al., 2019; Farrer et al., 2026; Ruiz-Ortega et al., 2024; Schoeps et al., 2020). These findings are particularly relevant during the transition to HE, when students may face increased emotional demands alongside academic and personal changes (Tuero et al., 2024; Zanden et al., 2018).

Despite these promising findings, previous EI intervention studies in HE have mostly considered university students as a broad population, with limited attention to interventions designed specifically for first-year students (Asiedu et al., 2025; Durlak et al., 2011; MacCann et al., 2020; Naylor et al., 2017; Wu and Gong, 2025). This is particularly relevant because the transition into HE represents a distinct developmental period with specific academic and personal challenges (Tuero et al., 2024; Zanden et al., 2018). Consequently, interventions designed for HE students in general may not address the specific needs associated with entering university. In addition, existing interventions often focus on general emotional competencies rather than explicitly integrating the challenges of academic adjustment, autonomy, and psychosocial adaptation during this transition (Asiedu et al., 2025; Geßler et al., 2021; MacCann et al., 2020).

Beyond this population-specific gap, the existing EI intervention literature shows substantial variation in how interventions are conceptualized, delivered, and evaluated (Asiedu et al., 2025; Geraci et al., 2026; Gong and Jiao, 2019; Mattingly and Kraiger, 2019). Previous reviews and meta-analyses have identified differences in theoretical models, intervention content, design characteristics, delivery procedures, and assessment approaches (Asiedu et al., 2025; Geraci et al., 2026; Gong and Jiao, 2019; Hodzic et al., 2018; Kotsou et al., 2019; Mattingly and Kraiger, 2019). These differences include the emotional abilities and competencies addressed, the number and duration of sessions, intervention activities and delivery formats, and the timing and tools used to assess outcomes; the theoretical rationale underlying interventions is not always clearly specified, and the competencies targeted are not consistently aligned with the measures used to evaluate change, potentially limiting conclusions regarding whether the intended emotional abilities have actually been developed (Asiedu et al., 2025; Geraci et al., 2026). Relevant information on intervention content, delivery, implementation procedures, and study methodology is also not always reported sufficiently, limiting replication, critical appraisal, and comparison across interventions (Asiedu et al., 2025; Gong and Jiao, 2019; Mattingly and Kraiger, 2019).

Collectively, the limited focus on first-year students and the variability in intervention design, content, and assessment make it difficult to determine how EI interventions should be tailored to the developmental and contextual needs of students entering HE. These gaps highlight the need for clearer guidance regarding the characteristics, competencies, content, and assessment strategies that should underpin EI interventions for this population.

This study employs a modified e-Delphi method to develop consensus-based guidelines for designing future ability-based EI interventions for first-year HE students to support emotional abilities associated with adaptation, engagement, and well-being. By integrating perspectives from an international panel of EI experts working in European and American Latin contexts, this study seeks to address the underrepresented Latin cultural contexts in international EI literature (Buettner et al., 2020; Crisp et al., 2015; Whitsed et al., 2024). Rather than proposing a universally applicable intervention framework, the study aims to provide culturally informed and context-sensitive recommendations grounded in expert consensus.

The Delphi method is a structured consensus-building technique that gathers expert judgment through iterative rounds of questionnaires, allowing the reconstruction of uncertain or incomplete information based on probabilistic reasoning and expert knowledge (Green, 2014; Jünger et al., 2017; Niederberger and Spranger, 2020). The inclusion of an international expert panel aimed to incorporate diverse professional and cultural perspectives relevant to EI intervention design in HE contexts (Barrios et al., 2020). Widely applied across program planning, assessment, policy development, and guideline formulation, Delphi studies have been particularly valuable in the development and content validation of interventions aimed at promoting well-being, health, mental health, and educational outcomes (Buettner et al., 2020; Santos et al., 2018; Whitsed et al., 2024).

The current study follows the Guidelines for Conducting and Reporting Delphi Studies (CREDES; Jünger et al., 2017) and is aligned with the United Nations Sustainable Development Goals (SDGs), specifically good health and well-being (SGD 3) and quality education (SDG 4) for all students. By fostering sustainable conditions for youth training and education, the program has the potential to improve access to employment and decent work while reducing inequalities (SDG8). Additionally, the study sought to consider cultural and contextual factors relevant to emotional expression, interpersonal relationships, and student adaptation in HE settings by including experts from culturally related contexts (Bernal et al., 2009).

## 2 Method

This study employed a modified e-Delphi methodology to develop consensus-based guidelines for an ability-based EI intervention protocol for first-year HE students. The Delphi method is particularly suitable for areas characterized by conceptual complexity and limited standardization, as it enables the systematic integration of expert judgment through iterative rounds of structured feedback (Beiderbeck et al., 2021; Jünger et al., 2017).

Quantitative data were obtained through Likert-scale, forced-choice, single-choice, and multiple-choice questions. Optional qualitative input was collected via open-ended response boxes, allowing participants to justify, expand on, or suggest modifications to the proposed intervention components. The comments provided during Round 1 were reviewed and, when applicable, informed the refinement of existing items and the development of additional questions or response options included in Round 2.

All surveys were conducted through an online platform (LimeSurvey). The use of an online and anonymous format aimed to facilitate independent expert judgment while minimizing potential dominance effects and social desirability influences commonly associated with face-to-face consensus procedures (Thompson, 2009). Participants provided informed consent to participate and agreed to maintain the confidentiality of the study process until the conclusion (see Supplementary material A). Privacy settings in Limesurvey were activated to ensure data privacy and protection. Informed consent, data collection, and data analysis procedures ensured participant anonymity and were conducted in accordance with the ethical requirements of the Declaration of Helsinki and international ethical guidelines for psychologists.

### 2.1 Participants

Experts were selected using a purposive sampling strategy commonly employed in Delphi studies involving specialized knowledge domains (Palinkas et al., 2015; Schifano and Niederberger, 2025). Potential participants were identified through professional networks, academic expertise mapping, publication records, and professional experience in the fields of EI, social and emotional learning, and psychological interventions in HE settings. Given the study’s focus on first-year HE students and EI intervention design, efforts were made to include participants with complementary expertise across clinical, educational, and research contexts, all with experience in the implementation of psychological interventions, familiarity with ability-based EI models, and broader socio-emotional learning (SEL) approaches. Experts were recruited from Latin countries to strengthen the ecological validity and implementation relevance of the intervention. The panel was selected based on the following criteria:

- Professional experience or scientific knowledge in implementing psychological interventions (educational or clinical) in educational settings;
- Authorship of relevant academic publications related to EI or other theories of social and emotional learning;
- Active scientific work in fields related to emotional intelligence, mental health, academic success, or higher education.

In line with recommendations for specialized Delphi expert panels, the final panel consisted of 12 experts, which falls within the range commonly considered appropriate for studies involving highly specialized expertise (Schifano and Niederberger, 2025). Invitations were emailed to 12 experts, including information about the study’s scope and objectives, the expected timeline for the rounds, the ethical requirements, and a brief description of the Delphi method along with instructions (see Supplementary material A). All invited experts agreed to participate and completed the informed consent process prior to inclusion in the study.

The panel consisted of 12 members (Supplementary material B, Table B1), with 75% being females, and a mean age of 44.92 years (SD = 10.54). They represented five different countries: Portugal (*n* = 4), Spain (*n* = 3), Italy (*n* = 2), Chile (*n* = 2), and Brazil (*n* = 1). Participants had professional backgrounds from clinical (50%), educational psychology (66.7%) backgrounds (see Table 1), with roles including researcher (*n* = 8), professor (*n* = 9), and psychologist with clinical practice (*n* = 7). Most participants reported expertise in promoting EI (*n* = 10), emotional competencies (*n* = 9), mental health (*n* = 10), and psychological interventions (*n* = 9). Additionally, 66.7% of the experts had more than 10 years of experience in their field, had authored more than 10 publications (66.7%), 50% held doctorate degrees, 33.3% held postdoctoral qualifications, and the remaining participants (16.7%) were completing doctoral training while holding a master’s degree.

**Table 1:** Consensual results from experts’ Likert-scale responses.

Dimensions	Subdimensions	Statements for agreement or importance ranking	Round	*M*	*SD*	% of agreement^*^
Intervention design	Activities between sessions	Include activities between sessions	1	3.92	1.51	75
Intervention content	EI abilities^+^	Perceiving Emotions	1	4.82	0.40	91.7
		Using Emotions		4.63	0.50	91.7
		Understanding Emotions		5	0.00†	91.7
		Managing Emotions		4.91	0.30	91.7

^+^Agreement percentages for this item were calculated using available responses only (*n* = 11), as one participant did not provide a response; “Round” indicates in which round the item was resolved; *“4” and “5” values on a 5-point Likert scale; †A standard deviation (SD) of 0 reflects the absence of variability in responses, indicating complete agreement among experts for this item.

### 2.2 Survey design

This study utilized a modified e-Delphi design to systematically develop and refine survey items related to the structure and content of an ability-based EI intervention (Beiderbeck et al., 2021; Schifano and Niederberger, 2025). Rather than implementing an entirely open-ended first round, the initial questionnaire was developed from existing evidence identified through a review of the published literature (e.g., Hodzic et al., 2018; Kotsou et al., 2019). This approach was considered appropriate given the substantial body of evidence available to inform the design and evaluation of ability-based EI interventions. The extracted evidence was synthesized into themes related to the intervention format, focusing specifically on design, intervention content, and participant characteristics, guided by the checklist for group-based interventions proposed by Borek et al. (2015). Figure 1 illustrates the development process of the survey, including item generation and reorganization, expert recruitment, a pilot study, and two Delphi rounds. Across these stages, items were refined, rewritten, or added based on expert feedback, culminating in consensus on the final set of questions.

**Figure 1:**
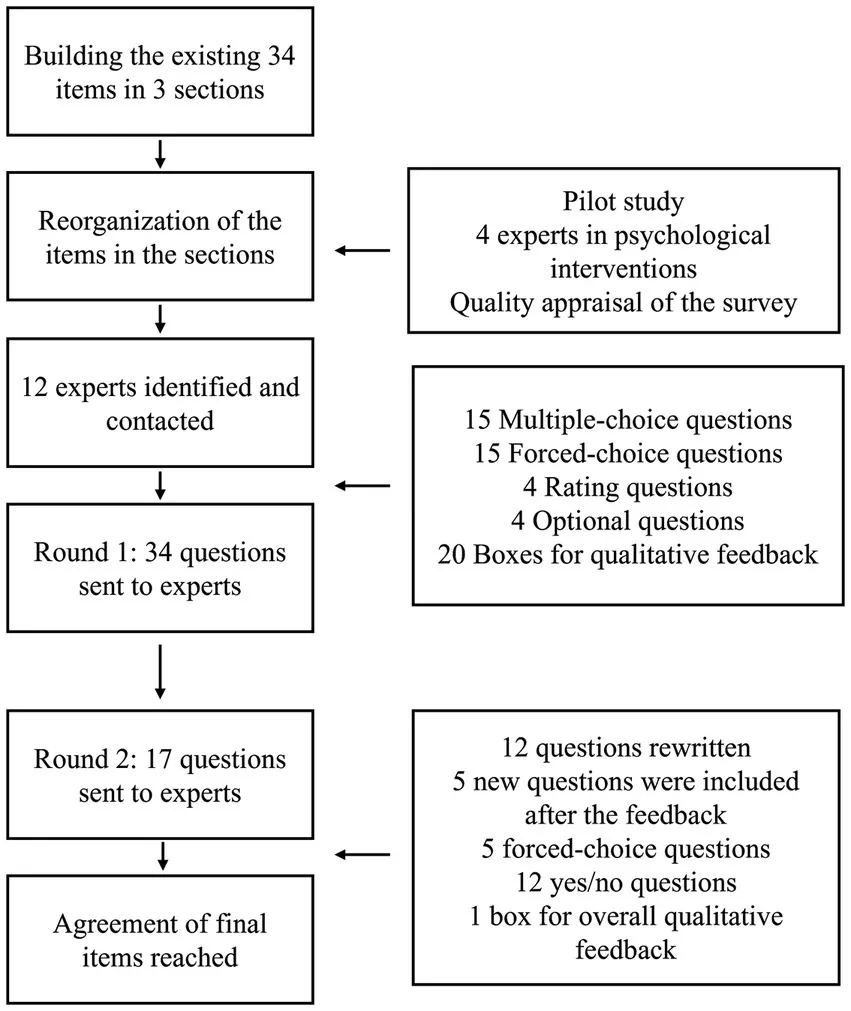
Flowchart of the Delphi study process.

Firstly, the survey was organized into three sections: (i) intervention design, which included questions about the number of group sessions, session length, frequency, total duration of interventions, materials used by participants, activities conducted during and after sessions, types of activities, group sizes, and the number of facilitators; (ii) intervention content, with questions focusing on the content of the sessions and the order in which sessions are sequenced; and (iii) efficacy assessment, containing questions regarding the assessment plan, the timing of assessments, and the assessment tools.

The predefined response options were developed based on evidence from systematic reviews addressing the effectiveness and methodological characteristics of EI interventions, while recurring methodological recommendations and gaps reported in the literature were used to inform the design and evaluation of future interventions (Hodzic et al., 2018; Kotsou et al., 2019; Mattingly and Kraiger, 2019), as well as EI assessment tools (Bru-Luna et al., 2021). We considered published studies on EI interventions for undergraduate students and adults aimed at enhancing well-being, mental health, and employability (e.g., Nelis et al., 2011; Schoeps et al., 2020), as well as literature focusing on EI interventions for adolescents (Cabello et al., 2016; Ruiz-Aranda et al., 2012; Ruiz-Aranda et al., 2013). Additionally, we reviewed previous research on academic success factors among undergraduate students (Araújo et al., 2019; MacCann et al., 2020; Zanden et al., 2018). In summary, the response options for the questions were shaped by the study’s objectives and a review of existing literature that addressed methodological considerations and recommendations for future interventions. For instance, regarding the question “number of sessions”, we included options for six or seven sessions, as previous studies frequently reported this range in HE and adult EI intervention contexts (Hodzic et al., 2018; Mattingly and Kraiger, 2019).

The answer options for the survey included multiple-choice, single-choice, and a 5-point Likert scale (where “1 = totally disagree” and “5 = totally agree”). Additionally, participants were allowed to provide open-ended responses for each survey item, enabling them to suggest options not included in the choices provided. When using this open-ended option, participants were encouraged to include bibliographic references that support their statements (e.g., “Do you suggest a different number of sessions? Please justify your choice and, if possible, add a reference to support it”).

The survey for the first round consisted of 22 main questions. A draft of the first round of the survey was piloted by four experts in psychological interventions, encompassing both academics and clinicians. These experts evaluated the survey for relevance, clarity of content, and any potential gaps or errors in its construction. Following the pilot phase, minor revisions were implemented to improve question clarity, reduce redundancy, and optimize the sequencing of survey items. These adjustments were intended to enhance comprehensibility and reduce respondent burden prior to the first Delphi round. Importantly, no substantial structural or conceptual changes were made to the overall survey framework following pilot testing.

### 2.3 Rounds

In the first round, the experts received a formal invitation via email, which included detailed information about the study, schedule, ethical requirements, and a link to complete the survey (see Supplementary material A). Participation began with obtaining informed consent, followed by the collection of sociodemographic and professional background data. Responses were collected over a two-week period from all recruited participants. Additionally, the expert panel was provided with contact details for the research team to facilitate clarification regarding study procedures or survey content throughout the Delphi process. No requests for additional clarification were received.

The first-round questionnaire included 34 items distributed across the three main domains of the intervention framework: intervention design, intervention content, and efficacy assessment. In addition to structured response options, participants were encouraged to provide qualitative feedback, suggest modifications, and propose additional intervention components through open-ended response boxes. Following completion of Round 1, all responses were reviewed by the research team. Items that did not achieve the predefined consensus threshold, together with recurring suggestions derived from the optional qualitative comments, informed the refinement of existing items and the development of additional items for Round 2. To enhance transparency, all comments and the corresponding decisions regarding item revision, addition, or retention were synthesized in a Supplementary material B, Table B3. No formal qualitative analysis was undertaken, as the purpose of the open-ended comments was to support the iterative refinement of the Delphi questionnaire rather than to generate qualitative findings.

A summary report containing aggregated results was sent after 1 week and was available for consultation for 3 weeks in a digital file. The file included graphics displaying the percentages of items that reached consensus, as well as those that proceeded to the second round due to a lack of consensus.

The second Delphi round was conducted using a more structured forced-choice format (e.g., yes/no responses) to facilitate clearer consensus decisions regarding items that remained unresolved after Round 1. A second email containing the instructions and link for the second-round survey was sent to all participants (see Supplementary material A). The Round 2 questionnaire consisted of 17 items and also included an optional open-ended commentary section for additional observations. All participants completed the second-round survey within 2 weeks. Upon completion of the Delphi process, participants received a final summary report containing the overall consensus results.

### 2.4 Consensus criteria

Consensus criteria were established *a priori* based on methodological recommendations from Delphi research involving specialized and relatively homogeneous expert panels, in which consensus thresholds commonly range between 50 and 95%, depending on study aims, the rating scale, and panel size (Schifano and Niederberger, 2025; Škrinjar et al., 2026). In the present study, consensus was defined as ≥ 60% agreement for forced-choice and multiple-choice questions, or a mean score of ≥3.5 on a 5-point Likert scale (with agreement indicated by scores of 4 or 5). In the present study, a nominal consensus threshold of 60% was specified *a priori*, in view of the exploratory aim of the study and the small, specialized expert panel (*N* = 12). The intention was to identify areas of convergence while avoiding an overly stringent criterion that might exclude potentially relevant areas of agreement in this panel. Given the panel size, this corresponded to a minimum of 8 experts, corresponding to 66.7% of agreement. This threshold was therefore interpreted as an indicator of preliminary or sufficient convergence rather than unequivocal consensus (e.g., Snape et al., 2014).

Participants were informed that predefined consensus criteria would guide item retention across rounds; however, the specific percentage threshold was not disclosed in order to minimize response bias during the consensus process.

Given the relatively small and specialized expert panel, items reaching 58.3% agreement (i.e., 7 out of 12 experts) were retained and reported separately as near-consensus findings when they addressed aspects directly relevant to the study aims. Although these items did not achieve the *a priori* consensus threshold, they differed from it by only one expert and were therefore interpreted cautiously and clearly distinguished from the expert consensus-based guidelines. They are presented as supplementary findings to provide additional context and inform future research.

### 2.5 Data analysis

Data were analyzed using Excel 16.0 and SPSS 30.0. Descriptive statistics (including percentages, means, and standard deviation) were calculated to summarize expert responses across Delphi rounds.

Consensus percentages were calculated based on the number of valid responses for each item. In cases of missing responses, analyses were conducted using available-case calculations, meaning that percentages reflected only the responses provided for the specific item under analysis. Missing responses were not treated as negative responses or abstentions in order to avoid artificially influencing agreement estimates. Qualitative responses obtained through optional open-ended questions in Round 1 were limited in number and did not undergo formal qualitative analysis. Instead, comments were reviewed by the research team to support item refinement and the development of additional questions for Round 2. This process was particularly relevant for dimensions that did not initially reach consensus, such as the number of intervention sessions, where expert comments informed additional response options and item clarification (see Supplementary material B, Table B3). Less frequent suggestions were retained as recommendations and reported descriptively. Convergence throughout the Delphi process was interpreted descriptively through agreement patterns observed across reassessed items presented in Tables 2–4 and Supplementary material B.

**Table 2:** Consensual results submitted to experts in rounds 1 and 2 for the intervention design dimension.

Subdimensions	Options	Round	*N*	% agreement
Total number of group sessions	^a^A total of 10 sessions: two per emotional ability (perception, use, understanding, management), an introductory session, and a final session	2	9	75
Length of group sessions	2 h	1	8	66.7
Frequency of group sessions	1 x week	1	11	91.7
Duration of the intervention	An intervention distributed in 1 semester	1	10	83.3
	First semester (September to January)		10	83.3
Missing sessions	^a^Can miss 1 session	2	9	75
Number of participants per group*	^a^Up to 15 participants (medium group)	2	11	91.7
Group composition criteria*	None	2	12	100
	Students’ schedules and availability		10	83.3
Number of facilitators	2 facilitators	1	8	66.7
Type of activities*	^a^The same amount of individual and group activities	2	9	75
Session activities*	Roleplays	1 and 2	11	91.7
	Class discussions		10	83.3
	Activities feedback		11	91.7
	Individual training tasks		7	58.3
	Team assignments		8	66.7
	Educational content (i.e., emotional literacy)		10	83.3
	Videos		9	75.5
	Case studies (presentation and discussion of real-life cases and solutions)		7	58.3
	Association between emotions and life events		8	66.7
	Display of emotional vocabulary		8	66.7
	Debates		7	58.3
	Games adapted to emotional content		9	75
	^a^Monitor emotions, thoughts, and experiences during the intervention		12	100
	^a^Relaxation exercises		11	91.7
Intervention materials*	General information about the intervention and objectives	1	11	91.7
	A support guide about the intervention and individual sessions		9	75
	Fact sheets summarizing each session		8	66.7
	Support material about session exercises		11	91.7
Activities between sessions (i.e., homework)*	Yes	1 and 2	12	100
	Specific prescribed activities		11	91.7
	^a^Worksheets for practice		12	100

“Round” indicates in which round the item was resolved; *Yes/ No response for including each option; ^a^item included only in Round 2.

**Table 3:** Consensual results submitted to experts in rounds 1 and 2 for the intervention content dimension.

Subdimensions	Options	Round	*n*	% agreement
Additional competencies*	Yes/ Include	1	8	66.7
	Personal competencies		9	75
	Mental health and well-being		9	75
Personal competencies*	Identity and self-esteem	1	7	58.3
	Interpersonal relationships		7	58.3
	Empathy		7	58.3
Mental health and well-being*	Well-being and self-care	1	8	66.7
	Stress management and coping strategies		9	75
	Mental health (i.e., anxiety and depression)		9	75
	Emotional regulation strategies		11	91.7
Academic success competencies*	Self-regulated learning	1	7	58.3
	The balance between academic, social, and personal life		7	58.3
	Relationship with peers		8	66.7
Sequence of the sessions^+^	Embed contents (i.e., when focusing on EI abilities, the sessions will apply the contents to students’ academic life and well-being)	1	10	83.3

^+^Agreement percentages for this item were calculated using available responses only (*n* = 11), as one participant did not provide a response; “Round” indicates in which round the item was resolved; *Yes/ No response for including each option; ^a^item included only in Round 2.

**Table 4:** Consensual results submitted to experts in rounds 1 and 2 for the intervention efficacy assessment dimension.

Subdimensions	Options	Round	*N*	% agreement
Methods for efficacy assessment	Mixed-methods	1	12	100
Quantitative assessment*	Self-report	1	11	91.7
	Ability tests		8	66.7
Qualitative assessment*	Individual interviews	1	7	58.3
	Focus group		9	75
EI assessment tools*	Trait Meta Mood Scale (TMMS; Fernandez-Berrocal and Extremera, 2004)	1	8	66.7
	Situational Test of Emotion Management (STEM; MacCann and Roberts, 2008)		7	58.3
Additional assessment measures*	Positive and Negative Affects Scale (PANAS; Watson et al., 1988)	1	10	83.3
	Satisfaction With Life Scale (SWLS; Diener et al., 1985)		7	58.3
	Rosenberg Self-Esteem Scale (Rosenberg, 1965)		8	66.7
Academic Success Assessment*	Academic GRADES	1	8	66.7
	Self-Regulation Questionnaire-Academic		8	66.7
Control variables*	Generalized self-efficacy	1 and 2	8	66.7
	Motivation for the intervention		8	66.7
	Scientific field		7	58.3
	Gender		11	91.7
	Age		10	83.3
	Family educational background		9	75
	^a^Mental health issues, such as depression and anxiety		10	83.3
	^a^Trait features (i.e., personality traits)		10	83.3
Pre-test assessment^+^	1 week before the intervention	1	9	75
Post-test assessment	-	-		
Follow-up moments	1 moment	1	8	66.7
Session assessment*	^a^Include quality appraisal after each session (i.e., assess the session)	1 and 2	10	83.3
	Level of satisfaction with the session		9	75
	Level of satisfaction with what was learned		8	66.7
	Level of perceived utility of the session		11	91.7
	Commentary section (e.g., suggestions for future sessions)		8	66.7

^+^Agreement percentages for this item were calculated using available responses only (*n* = 11), as one participant did not provide a response; “Round” indicates in which round the item was resolved; *Yes/ No response for including each option; ^a^item included only in Round 2.

## 3 Results

The results are organized into three sections following the survey structure: (i) intervention design, (addressing the number, length, frequency, and duration of sessions, as well as the materials used by participants), activities conducted during and after sessions, types of activities, group sizes, and the number of facilitators involved; (ii) intervention content, focusing on the content of each session and the order in which sessions are sequenced; and (iii) efficacy assessment, covering the assessment plan, including the time points and tools used for assessment.

All 12 experts completed both Delphi rounds, with no participant attrition throughout the study. One participant (8.3%) did not respond to three item sets during Round 1 related to EI abilities, session sequence, and pre-test assessment. This particular participant reported not feeling sufficiently experienced to provide informed responses for these domains and therefore chose not to answer these items. Consequently, analyses for these items were conducted using the number of valid responses available for each item.

Results are presented according to items reaching the predefined consensus criteria, while items approaching consensus (58.3% agreement) are reported separately as near-consensus findings to provide supplementary information for future research and should not be interpreted as expert consensus-based guidelines.

### 3.1 Intervention design

Round 1 resulted in an agreement on eight items that could be included in the EI intervention protocol guidelines (Tables 1, 2). The experts agreed on the following intervention design elements: two-hour group sessions (66.7%), scheduled once a week (91.7%), over one semester (83.3%), preferably in the first semester (September to January; 83.3%), and delivered preferably by two facilitators (66.7%). For session activities, consensus was reached on several activities, with approval ranging from 66.7 to 91.7%. These activities included role plays, class discussions, feedback activities, team assignments, educational content (i.e., emotional literacy/vocabulary), videos, associations between emotions and life events, displays of emotional vocabulary, and games adapted to emotional concepts. Additionally, a small number of activities reached near-consensus levels (58.3% agreement), including individual training tasks, case studies, and debates. Regarding the materials to be used in the sessions, participants should receive general information about the intervention and its objectives (91.7%), a support guide for the intervention and individual sessions (75%), fact sheets summarizing each session (66.7), and support materials for session exercises (91.7%). The experts also agreed to include specific prescribed activities between sessions, provided they are related to the sessions (91.7%).

Questions regarding the number of sessions, group composition, missing sessions, type of activities, and the number of participants per group did not reach a consensus in the first round and were revised for a second round of review (Supplementary material B, Table B2). This process involved adjustments to the questions’ format (e.g., changing 5-point Likert scale questions to yes/no questions) and contents, incorporating additional options suggested by experts (Supplementary material B, Table B3), and rewriting questions for clarity (e.g., activities between sessions).

In round 2, all subdimensions achieved consensus (Table 2). Experts reached consensus on EI interventions with a total of 10 sessions: two for each emotional ability (perception, use, understanding, and management), along with an introductory session and a final session (which received 75% agreement). Experts also agreed that participants may miss one session of the intervention (75% agreement) without being excluded from the efficacy assessment study. Groups should consist of up to 15 participants (91.7% agreement) and should be formed according to students’ self-declared available schedules (83.3% agreement).

Experts agreed that the intervention should include the same amount of individual and group activities (75% agreement), incorporate activities between sessions (100% agreement), and integrate emotional screenings and relaxation exercises during the sessions (100% agreement). Additionally, participants should receive worksheets for practice related to the sessions (100%).

In sum, the consensus-derived guidelines for the design of an EI intervention tailored for first-year HE students indicated that the intervention should consist of a total of 10 sessions, with two sessions dedicated to each emotional ability (perception, use, understanding, and management), as well as an introductory session and a concluding session. Each session should last 2 h and be scheduled once a week over the course of one semester, preferably the first semester, allowing participants the flexibility to miss one session. To maintain an effective environment, groups should be limited to 15 participants and organized based on students’ schedules and availability. The program should be facilitated by two specialists. Consensus was also reached on several group-based and psychoeducational activities, including role-plays, class discussions, emotional vocabulary exercises, feedback activities, and team assignments. In addition, several activities approached but did not reach the predefined consensus threshold (58.3% agreement), namely case studies, debates, and individual training tasks. Participants should also receive comprehensive information about the intervention, including details on the sessions, practice worksheets, and activities to complete between sessions that align with the intervention content (Table 2).

### 3.2 Intervention content

Regarding the intervention’s contents, a stronger consensus was observed for core emotional competencies than for broader academic-related competencies (Tables 1, 3). Experts agreed that emotional abilities hold varying levels of importance within the intervention (e.g., perceiving emotions vs. understanding emotions; see Table 1). Additionally, experts agreed that competencies related to personal development (75%) and mental health and well-being (75%) should be included (66.7% overall agreement). Several personal-development-related competencies approached but did not reach the predefined consensus threshold (58.3% agreement): identity and self-esteem, interpersonal relationships, and empathy. Experts also supported the integration of mental health and well-being-related themes within the intervention framework, particularly self-care (66.7%), stress management and coping strategies (75%), mental health literacy (75%), and emotion regulation strategies (91.7%).

The broader domain of academic success competencies did not reach the predefined consensus threshold (50%) and, therefore, was not retained as a formal intervention component. However, some individual subdimensions within this domain achieved higher levels of agreement, including relationships with peers (66.7%), near-consensus levels for self-regulated learning (58.3%), and balance among academic, social, and personal life (58.3%).

Additionally, experts agreed (83.3%) that, when selected beforehand, these topics should be embedded into the intervention by contextualizing EI abilities within students’ academic experiences and overall well-being. The relevance of these competencies within the intervention framework was further reflected in the experts’ qualitative comments (Supplementary material B, Table B3).

In summary, experts reached consensus on including the four EI abilities in the intervention framework, with understanding emotions perceived as particularly relevant. In addition to core emotional abilities, experts also supported the inclusion of several personal-development and mental health-related themes embedded within the intervention, including self-care, coping strategies, stress management, mental health literacy, and emotion regulation approaches. Academic competencies were not retained as an independent intervention domain due to the lack of formal consensus; however, certain academically related dimensions were considered potentially relevant as contextual elements in students’ adaptation experiences. Furthermore, several competencies related to identity, empathy, and interpersonal relationships approached, but did not reach the predefined consensus threshold and are therefore reported as near-consensus findings requiring cautious interpretation.

### 3.3 Efficacy assessment

Broad agreement was observed across most efficacy assessment dimensions during Round 1 (Table 4), particularly regarding the use of multimethod assessment approaches. Experts unanimously agreed that the assessment of intervention efficacy should adopt a mixed-methods approach (100%), with self-report measures (91.7%) and ability tests (66.7%) for quantitative assessments. Focus groups reached the predefined consensus threshold (75%), whereas individual interviews approached consensus levels (58.3%) and were therefore retained as provisional qualitative assessment strategies. Suggestions for the assessment of EI included the Trait Meta Mood Scale (TMMS; Fernandez-Berrocal and Extremera, 2004; 66.7%) and additional instruments approached, but did not reach, the predefined consensus threshold, including the Situational Test of Emotion Management (STEM; 58.3%) and the Satisfaction with Life Scale (SWLS; 58.3%). These measures were retained as provisional assessment options.

To assess the content of the intervention, experts suggested additional measures, including the Positive and Negative Affects Scale (PANAS; Watson et al., 1988; 83.3%), the Rosenberg Self-Esteem Scale (Rosenberg, 1965; 66.7%), and possibly the Satisfaction with Life Scale (SWLS; Diener et al., 1985; 58.3%). Academic success could also be evaluated using the Grade Point Average (GPA; 66.7%) and the Self-Regulation Questionnaire-Academic (Ryan & Connell, 1989; 66.7%). Further options can be found in Supplementary material B, Table B3.

For control variables to be considered when evaluating the intervention’s efficacy, the experts recommended the following factors: general self-efficacy (66.7%), motivation for the intervention (66.7%), gender (91.7%), age (83.3%), family background (75%), and the academic program’s scientific field (58.3%). Additional options for the assessment of psychopathological symptoms, such as depression and anxiety, and trait features (i.e., personality traits) were also included in Round 2 (Table 2) based on experts’ suggestions (Supplementary Table B3), and both reached a consensus (83.3%).

Finally, experts deemed that the pre-test assessment should take place 1 week before the intervention (75%) and the efficacy assessment may consist of only one post-intervention moment (66.7%). Additionally, the assessment of the session can include various components: the level of satisfaction with the session (75%), what was learned (66.7%), the perceived utility of the session (91.7%), and comments or suggestions for future interventions (66.7%). For clarity, the dimension addressing the session’s assessment was revised in Round 2, changing from “session assessment” to “quality appraisal” after each session to ensure the focus is on evaluating the session itself rather than on assessing the participant (Table 4). Throughout the Delphi rounds, experts did not reach consensus regarding the optimal timing for post-intervention assessment. Given that consensus had been achieved across the remaining dimensions and considering the stability of responses regarding this item across rounds, an additional Delphi round was not conducted.

In summary, experts supported the use of a mixed-methods efficacy assessment approach combining quantitative and qualitative strategies. Consensus favored the inclusion of both self-report and ability-based EI measures (e.g., Trait Meta-Mood Scale), alongside qualitative approaches such as focus groups. Several additional assessment tools and qualitative procedures approached but did not reach the predefined consensus threshold, and are therefore reported as near-consensus findings to inform future research. Experts also emphasized the importance of assessing broader indicators related to student well-being, emotional functioning, and academic adjustment, while considering relevant sociodemographic and contextual control variables. Although experts agreed on the importance of including at least one post-intervention assessment point, no consensus was reached regarding the optimal timing of follow-up assessment. One follow-up moment was considered suitable for the evaluation.

The resulting document, Guidelines for an Emotional Intelligence Intervention for First-Year Higher Education Students (Supplementary material C), presents the recommendations that achieved the pre-specified Delphi consensus threshold. For transparency, items approaching consensus are also included and clearly identified as near-consensus findings.

## 4 Discussion

This Delphi study developed consensus-derived guidelines for an ability EI intervention framework for first-year HE students, integrating perspectives from experts from five different countries and diverse professional backgrounds, with a strong understanding of EI development. The findings contribute to ongoing efforts to reduce heterogeneity in the design, content, and assessment procedures of EI interventions reported in previous literature (Gong and Jiao, 2019; Hodzic et al., 2018; Kotsou et al., 2019; Mattingly and Kraiger, 2019), while offering a structured and context-sensitive framework to guide the design and implementation of future interventions in HE settings.

### 4.1 Intervention design

The proposed guidelines suggest that future interventions following an ability-based EI model should be structured into 10 weekly sessions of approximately 2 h, distributed across one semester, preferably during the first semester of students’ university experience. Previous EI interventions in HE have used different durations and frequencies, ranging from shorter interventions to more extended, multi-session programs (e.g., Asiedu et al., 2025). This variability is consistent with the heterogeneity reported in reviews of EI interventions (Gong and Jiao, 2019; Mattingly and Kraiger, 2019), suggesting that no single intervention dose has yet been established as optimal. The panel’s preference for the intervention’s implementation during the first semester is particularly relevant given that this period represents an initial phase of adjustment to HE, during which students are simultaneously navigating new academic and personal challenges, as well as changes in their social environment (Tuero et al., 2024; Zanden et al., 2018). Delivering the intervention early in the transition may therefore provide the timely support needed to navigate these challenges. The present findings provide an expert-informed structure that balances sufficient exposure to EI training with the practical demands of the academic context.

The panel further agreed that the allocation of intervention time should reflect the complexity of the specific abilities being trained. Accordingly, more complex abilities, such as understanding emotions, which involves knowledge of the characteristics and interrelationships of emotions and the ability to predict emotional scenarios, and managing emotions, which involves regulating one’s own emotions and influencing others’ emotions, were considered to require greater intervention time, whereas perceiving emotions, which focuses on developing emotional vocabulary and identifying emotions, may require less time (Geßler et al., 2021; Joseph and Newman, 2010; Mayer et al., 2016; Salovey and Mayer, 1990). This recommendation is consistent with the sequential development of EI proposed within the ability model and the cascading model, whereby more complex emotional abilities build upon more foundational processes (Joseph and Newman, 2010; Mayer et al., 2016). It is also aligned with Social and Emotional Learning (SEL) approaches, which emphasize the sequential and cumulative development of socioemotional competencies (Durlak et al., 2011). In this respect, the present Delphi findings provide expert consensus supporting the view that EI abilities may require different levels of intervention duration according to their relative complexity, reinforcing the importance of a differentiated and sequential approach to EI training.

The panel also emphasized the use of active and experiential methodologies, including role-plays, case studies, discussions, debates, individual tasks, and group activities. The recommendation for a balance between individual and group activities further reflects the need to combine opportunities for individual reflection and ability practice with interpersonal learning experiences. These recommendations are consistent with previous EI interventions that have incorporated role-play, group activities, discussion, and reflective exercises to facilitate the practical application of emotional competencies (Mattingly and Kraiger, 2019). They also align with experiential and social cognitive approaches, which emphasize learning through active participation, reflection, and application to meaningful contexts (Bandura et al., 1997; Kolb and Boyatzis, 1970). Although previous interventions have used diverse combinations of activities and delivery strategies, and the available evidence does not establish a single activity format as superior (Asiedu et al., 2025; Mattingly and Kraiger, 2019), the present findings suggest the implementation of a combination of active methodologies while allowing flexibility in their specific application. The consensus regarding tailored intervention materials and structured activities between sessions further emphasizes the importance of extending EI practice beyond the group sessions. Providing information about the intervention and its objectives, session guides, fact sheets, exercise materials, and prescribed activities or worksheets may support continued practice and reflection in students’ everyday contexts (Durlak et al., 2011; Mattingly and Kraiger, 2019).

The practical implementation of a 10-session EI intervention within HE contexts also raises important feasibility considerations. Implementation research emphasizes that the success of an intervention depends not only on its effectiveness, but also on whether it can be appropriately and feasibly implemented within a given setting, with factors such as acceptability, appropriateness, feasibility, fidelity, implementation costs, and sustainability requiring consideration (Proctor et al., 2023). In the present study, experts recommended groups of up to 15 students, two facilitators, and taking into account students’ schedules and availability when composing the intervention groups, reflecting the need to balance intervention structure with the practical demands of HE settings. The possibility of missing one session may also provide some flexibility within the academic demands of first-year students, allowing students to successfully complete the intervention. Although experts supported a relatively comprehensive intervention structure, universities may face practical challenges related to scheduling constraints, participant retention, facilitator availability, and institutional resource allocation. Consequently, the sustainability of such interventions may depend not only on their content and structure, but also on how they are integrated into the broader academic environment, including interpersonal and contextual factors that may influence engagement and sustained participation (Proctor et al., 2023). Future implementation should therefore remain sensitive to institutional and cultural variability across HE settings, with adaptations in scheduling, delivery format, facilitator training, and student support structures where necessary. Additionally, the cultural context may also be relevant when interpreting the present findings, since the inclusion of experts from Latin countries in Europe and Latin America brought professional experience from diverse cultural and educational contexts, contributing to the contextual framing of the proposed intervention, as recommended by prior research (e.g., Bernal et al., 2009; Crisp et al., 2015).

### 4.2 Intervention content

The intervention was developed around the four EI abilities—perceiving, using, understanding, and managing emotions—as its theoretical and conceptual core, based on the ability EI model (Mayer et al., 2016; Salovey and Mayer, 1990). The Delphi process was subsequently used to identify and refine additional competencies and themes that could be incorporated into the intervention content. Previous EI interventions in HE have varied in their theoretical scope, with some adopting mixed-model approaches that incorporate broader personal, social, and well-being-related competencies (Asiedu et al., 2025; Gilar-Corbí et al., 2018; MacCann et al., 2020). Although such approaches have reported beneficial outcomes, systematic reviews have also highlighted substantial heterogeneity and less consistent findings across interventions using broader mixed-model frameworks (Gong and Jiao, 2019; Hodzic et al., 2018; Kotsou et al., 2019; Mattingly and Kraiger, 2019). In this context, the present findings support maintaining the four EI abilities as the core framework while using additional themes and competencies to contextualize their development and application. These included identity and self-esteem, empathy, interpersonal relationships, well-being and self-care, stress management and coping, mental health, and emotional regulation strategies. Rather than representing additional EI competencies, these themes were understood as contextualized applications through which EI abilities could be practiced and transferred to students’ everyday academic and interpersonal experiences. This approach preserves the theoretical coherence of the ability EI model while increasing the ecological relevance of the intervention for first-year HE students, particularly during a period characterized by changes in academic demands, social relationships, autonomy, and personal well-being (Araújo et al., 2019; Tuero et al., 2024; Zanden et al., 2018; Wu and Gong, 2025).

Furthermore, the lack of consensus regarding the inclusion of academic success competencies as a broader intervention domain is noteworthy. In the present study, although the broader academic success competencies domain did not achieve consensus, one of its specific dimensions, namely relationships with peers, received sufficient agreement to be retained. One possible explanation is that the notion of academic success may be perceived as relatively instrumental or performance-oriented when it is primarily associated with outcomes such as study strategies, academic performance, or self-regulated learning. Such content may be viewed as less directly aligned with the primary purpose of an EI intervention than competencies involving interpersonal and emotional functioning. On the other hand, relationships with peers may provide a more direct context for the development and application of emotional abilities, particularly those related to perceiving, understanding, and managing emotions. This interpretation is consistent with the interpersonal dimension of EI and with evidence linking EI to social functioning and interpersonal adjustment in educational contexts (Asiedu et al., 2025; MacCann et al., 2020; Mayer et al., 2016). Thus, the findings may indicate that, within an EI-focused intervention, academic experiences are more readily incorporated when they provide meaningful relational or emotional contexts rather than when they are framed primarily in terms of academic performance or instrumental skills.

### 4.3 Intervention assessment

The assessment plan derived from this Delphi study emphasizes a mixed-methods approach, combining quantitative and qualitative methods to capture different aspects of intervention outcomes. Quantitative assessment included both self-report measures and ability tests, while qualitative assessment involved focus groups and, when appropriate, individual interviews. The combination of these approaches may provide complementary information on changes in emotional abilities and self-perceived competencies, as well as participants’ experiences of the intervention. The inclusion of qualitative methods is also relevant from an implementation perspective, as interviews and focus groups are often used to provide information on the acceptability, appropriateness, and feasibility of the intervention, complementing quantitative assessments of intervention outcomes (Proctor et al., 2023). This approach is consistent with previous EI intervention research highlighting the value of combining different assessment methods when evaluating changes in emotional competencies (Hodzic et al., 2018; Kotsou et al., 2019).

For the assessment of EI, the panel suggested combining self-report and ability-based measures, specifically the Trait Meta-Mood Scale (TMMS; Fernandez-Berrocal and Extremera, 2004) and the Situational Test of Emotion Management (STEM; MacCann and Roberts, 2008). These measures are consistent with assessment approaches commonly used in international EI research (Bru-Luna et al., 2021), while also reflecting the theoretical orientation of the proposed intervention. The use of measures based on different assessment approaches is particularly relevant within an ability-based EI intervention, as self-report instruments primarily capture individuals’ perceptions of their emotional abilities, whereas ability-based measures assess performance in emotion-related tasks (MacCann et al., 2020; Mayer and Caruso, 2025). Accordingly, combining these approaches may provide a more differentiated assessment of intervention-related changes than relying on a single type of measure. Importantly, however, the selection of assessment measures should not rely solely on their established use in EI research but should also reflect their conceptual alignment with the content and objectives of the intervention, as previous reviews have identified insufficient alignment between intervention content and outcome assessment as a limitation of EI intervention studies (Asiedu et al., 2025; Geraci et al., 2026).

The panel further recommended brief assessments at the end of each session, covering satisfaction with the session and with the learning achieved, perceived utility, and open comments or suggestions. These process measures can complement the pre- and post-intervention assessments by capturing participants’ immediate perceptions of the intervention and identifying aspects that may be refined during future implementation. Such information is also relevant to implementation research, where participants’ experiences can inform the acceptability and appropriateness of an intervention (Proctor et al., 2023).

Several additional variables, including generalized self-efficacy, intervention motivation, personality traits, and relevant demographic and mental health characteristics, were included because they are frequently examined in EI research and have also been considered in previous EI intervention studies (Asiedu et al., 2025; Geraci et al., 2026; Schoeps et al., 2020). Although these variables are not primary intervention outcomes, their inclusion may provide additional information for interpreting intervention findings and can be considered in future studies according to their specific research questions and design.

Regarding assessment timing, the panel of experts agreed that a pre-test 1 week before the intervention provides an adequate baseline (Kotsou et al., 2019; Schoeps et al., 2020). In contrast, no consensus emerged regarding the optimal timing of post-intervention assessment. This lack of consensus is consistent with the variability reported in previous EI intervention research, in which assessment schedules have differed across studies, and the timing of outcome assessment has not been standardized (Gong and Jiao, 2019; Hodzic et al., 2018; Kotsou et al., 2019). This variability may also reflect differences in the expected temporal development of emotional abilities: although changes in emotional knowledge and self-perceived competencies may be detected shortly after training, the transfer and application of emotional abilities to everyday contexts may require additional time (Gong and Jiao, 2019; Hodzic et al., 2018; Kotsou et al., 2019). Consequently, the absence of consensus regarding post-intervention timing appears to reflect the broader heterogeneity of assessment practices and the uncertainty regarding when intervention-related changes are most appropriately captured. Although no formal consensus was achieved regarding the optimal timing of post-intervention assessment within the Delphi process, we propose that conducting the post-intervention assessment approximately 1 week after program completion may represent a methodologically coherent option when baseline assessment is conducted 1 week prior to intervention onset. This proposed interval is intended to provide a brief period between intervention completion and outcome assessment, allowing participants some time to reflect upon and integrate the emotional learning and experiences developed throughout the intervention.

Finally, the panel also recommended including at least one follow-up assessment time point. However, no specific timing was established, reflecting the lack of clear evidence regarding the most appropriate point at which to assess the longer-term effects of EI interventions. Previous research suggests that emotional abilities may continue to develop and be applied beyond the immediate intervention period, but the timing of these changes may vary across interventions and contexts (Gong and Jiao, 2019; Hodzic et al., 2018; Kotsou et al., 2019). Therefore, rather than prescribing a specific follow-up interval, future researchers should determine the timing of follow-up assessments according to the objectives, design, and context of their studies.

### 4.4 Limitations and strengths of the study

Several limitations should be considered when interpreting the findings of this study. First, although the Delphi methodology is particularly useful for generating expert-informed recommendations in emerging or heterogeneous fields, the resulting guidelines remain based on expert consensus rather than empirical validation. Consequently, the present findings should be interpreted as a preliminary, consensus-derived intervention framework that requires future pilot testing, implementation studies, and longitudinal evaluation before broader application in HE settings.

Second, the current study relied on a purposive sampling strategy involving experts identified through professional and academic expertise networks. Although this approach is consistent with the Delphi methodology for specialized panels, it may increase the possibility of selection bias and shared theoretical perspectives among participants. In addition, the relatively small sample size and concentration of experts from Latin European and Latin American contexts may limit the transferability of findings to other cultural, institutional, or educational systems. Similarly, the predominance of participants with academic and research backgrounds may also have reduced the representation of perspectives from other applied contexts, such as university counseling services or broader student support systems. Moreover, although the panel included substantial expertise in EI, mental health, and psychology interventions, fewer experts reported specific experience in designing and implementing interventions for first-year HE students. Future studies would benefit from involving multidisciplinary expert panels with complementary expertise to further strengthen the contextual relevance and practical applicability of the resulting guidelines.

Another limitation concerns the lack of consensus regarding the optimal timing of post-intervention assessment. This disagreement may reflect broader conceptual and methodological heterogeneity within the field of EI intervention research, particularly concerning the temporal development and stabilization of emotional abilities following training. Furthermore, although near-consensus findings are reported because they may inform future research and intervention development, they should be interpreted with caution and not be afforded the same weight as recommendations reaching the predefined consensus threshold.

Despite these limitations, the study has several strengths. The findings contribute to the growing effort to develop more structured and theoretically coherent EI intervention frameworks for first-year HE students, a population still underrepresented in the EI intervention literature. The integration of experts from multiple Latin cultural contexts allowed the study to incorporate perspectives relevant to emotional expression, interpersonal dynamics, and adaptation processes that may differ from the predominantly Anglo-Saxon contexts represented in much of the existing literature (Crisp et al., 2015; Whitsed et al., 2024). Methodologically, the study combines expert opinion with a systematic, data-driven approach, helping to reduce bias and improve the relevance of the findings for intercultural interventions. This approach contributed to the development of a context-sensitive and consensus-informed intervention framework while helping to address some of the heterogeneity identified in previous reviews of EI interventions. By responding to gaps highlighted in prior meta-analytic literature, the study contributes with preliminary guidelines that may support the future development, adaptation, and evaluation of EI interventions across diverse HE contexts.

### 4.5 Implications for research and practice

Future research should explore how the developed guidelines operate in real HE settings, particularly regarding feasibility, implementation processes, participant engagement, and contextual adaptability. Additional studies are also needed to explore how EI interventions grounded in the ability model relate to students’ emotional adjustment, academic adaptation, and well-being across different stages of the HE experience. Longitudinal and cross-cultural research may further contribute to understanding how emotional abilities develop and are applied over time in diverse educational contexts. In this sense, future pilot studies and implementation research will be essential for refining, adapting, and empirically evaluating the proposed intervention framework.

Expanding the proposed intervention framework to different student populations, academic stages, and institutional contexts may also help clarify the extent to which these recommendations remain applicable across diverse HE environments. In particular, future studies could further explore how contextual variables, cultural norms surrounding emotional expression, and institutional support structures influence the implementation and reception of EI interventions.

From a practical perspective, HE institutions may consider integrating emotional support initiatives into student counseling, adaptation, and well-being services, particularly during periods of academic transition. Beyond student-centered initiatives, faculty training is essential to foster a supportive learning environment and ensure early and consistent EI-based support throughout students’ academic and personal development. Furthermore, the use of structured and multimethod assessment strategies may support more systematic monitoring of intervention implementation and student experiences over time.

In conclusion, this study presents expert consensus-based intervention guidelines to guide the design and implementation of ability-based EI interventions for first-year HE students. By integrating perspectives from experts across multiple Latin cultural contexts, the study contributes with context-sensitive recommendations that may inform future research, pilot implementation, and the continued development of EI interventions in HE settings.

## Data Availability

The original contributions presented in the study are included in the article/Supplementary material, further inquiries can be directed to the corresponding author.

## References

[ref1] Acebes-Sánchez J. García-Naveira A. Conners R. T. García-Merino S. (2025). Emotional intelligence as a predictor of mental health in undergraduate students. BMC Psychol. 13:1130. doi: 10.1186/s40359-025-03241-7, 41074152 PMC12512798

[ref2] Amaro P. Fonseca C. Afonso A. Jacinto G. Gomes L. Pereira H. . (2024). Depression and anxiety of Portuguese university students: a cross-sectional study about prevalence and associated factors. Depress. Anxiety 2024:5528350. doi: 10.1155/2024/5528350, 40226682 PMC11919063

[ref3] Araújo A. M. Gomes C. M. A. Almeida L. S. Nunez J. C. (2019). A latent profile analysis of first-year university students’ academic expectations. An. Psicol. 35, 58–67. doi: 10.6018/analesps.35.1.299351

[ref4] Arnett J. J. Žukauskienė R. Sugimura K. (2014). The new life stage of emerging adulthood at ages 18-29 years: implications for mental health. Lancet Psychiatry 1, 569–576. doi: 10.1016/S2215-0366(14)00080-7, 26361316

[ref5] Asiedu A. Emslander V. Müller-Kreiner C. Seidel T. Holzberger D. (2025). How to promote emotional intelligence among university students: a research synthesis. Z. Psychol. 233, 93–109. doi: 10.1027/2151-2604/a000594

[ref9001] Baik C. Larcombe W. Brooker A. (2019). How universities can enhance student mental wellbeing: The student perspective. High. Educ. Res. Dev. 38:674–687. doi: 10.1080/07294360.2019.1576596

[ref6] Bandura A. Freeman W. H. Lightsey R. (1997). Self-efficacy: the exercise of control. J. Cogn. Psychother. 13, 158–166.

[ref7] Barrios M. Guilera G. Nuño L. Gómez-Benito J. (2020). Consensus in the Delphi method: what makes a decision change? Technol. Forecast. Soc. Change 158:120484. doi: 10.1016/j.techfore.2020.120484

[ref8] Beiderbeck D. Frevel N. von der Gracht H. A. Schmidt S. L. Schweitzer V. M. (2021). Preparing, conducting, and analyzing Delphi surveys: cross-disciplinary practices, new directions, and advancements. MethodsX 8:101401. doi: 10.1016/j.mex.2021.101401, 34430297 PMC8374446

[ref9] Bernal G. Jiménez-Chafey M. I. Domenech M. (2009). Cultural adaptation of treatments: a resource for considering culture in evidence-based practice. Prof. Psychol. Res. Pr. 40, 361–368. doi: 10.1037/a0016401

[ref10] Borek A. Abraham C. Smith J. R. Greaves C. J. Tarrant M. (2015). A checklist to improve reporting of group-based behavior-change interventions. BMC Public Health 15:963. doi: 10.1186/s12889-015-2300-6 PMC4583168 26403082

[ref11] Bru-Luna L. M. Martí-Vilar M. Merino-Soto C. Cervera-Santiago J. L. (2021). Emotional intelligence measures: a systematic review. Health 9:1696. doi: 10.3390/healthcare9121696, 34946422 PMC8701889

[ref12] Buettner D. Nelson T. Veenhoven R. (2020). Ways to greater happiness: a Delphi study. J. Happiness Stud. 21, 2789–2806. doi: 10.1007/s10902-019-00199-3

[ref13] Cabello R. Castillo R. Rueda P. Fernández-Berrocal P. (2016). Programa INTEMO+: Mejorar la inteligencia emocional de los adolescentes. Madrid: Ediciones Pirámide.

[ref14] Crisp G. Taggart A. Nora A. (2015). Undergraduate Latina/o students: a systematic review of research identifying factors contributing to academic success outcomes. Rev. Educ. Res. 85, 249–274. doi: 10.3102/0034654314551064

[ref9002] Diener E. Emmons R. A. Larsen R. J. Griffin S. (1985). The Satisfaction With Life Scale. J. Pers. Assess. 49:71–75. doi: 10.1207/s15327752jpa4901_13, 16367493

[ref15] Durlak J. A. Weissberg R. P. Dymnicki A. B. Taylor R. D. Schellinger K. B. (2011). The impact of enhancing students’ social and emotional learning: a meta-analysis of school-based universal interventions. Child Dev. 82, 405–432. doi: 10.1111/j.1467-8624.2010.01564.x, 21291449

[ref9003] Farrer L. M. Jackson H. M. Gulliver A. Calear A. L. Batterham P. J. (2026). Mental health among first-year students transitioning to university in Australia: A longitudinal study. Psychol. Rep. 129:2861–2882. doi: 10.1177/00332941241295978, 39440480 PMC13287367

[ref16] Fernandez-Berrocal P. Extremera N. (2004). Validity and reliability of the Spanish modified version of the trait meta-mood scale. Psychol. Rep. 94, 751–755. doi: 10.2466/pr0.94.3.751-755, 15217021

[ref17] Fruehwirth J. C. Biswas S. Perreira K. M. (2021). The Covid-19 pandemic and mental health of first-year college students: examining the effect of Covid-19 stressors using longitudinal data. PLoS One 16:e0247999. doi: 10.1371/journal.pone.0247999, 33667243 PMC7935268

[ref18] Geraci A. Scifo L. D’Amico A. Fernández-Berrocal P. Cabello R. (2026). Emotional intelligence training programs for teachers: a systematic review. Soc. Psychol. Educ. 29:84. doi: 10.1007/s11218-026-10255-5

[ref19] Geßler S. Nezlek J. B. Schütz A. (2021). Training emotional intelligence: does training in basic emotional abilities help people to improve higher emotional abilities? J. Posit. Psychol. 16, 455–464. doi: 10.1080/17439760.2020.1738537

[ref9004] Gilar-Corbí R. Pozo-Rico T. Sánchez B. Castejón J. L. (2018). Can emotional competence be taught in higher education? A randomized experimental study of an emotional intelligence training program using a multimethodological approach. Front. Psychol. 9:1039. doi: 10.3389/fpsyg.2018.01039, 29997545 PMC6030676

[ref20] Gong Z. Jiao X. (2019). Are effect sizes in emotional intelligence field declining? A meta-meta analysis. Front. Psychol. 10:1655. doi: 10.3389/fpsyg.2019.01655, 31379681 PMC6646523

[ref21] Green R. A. (2014). The Delphi technique in educational research. SAGE Open 4:2158244014529773. doi: 10.1177/2158244014529773

[ref22] Hashim M. A. Tlemsani I. Ndrecaj V. Cockrill A. (2025). Modelling the impact of emotional intelligence on academic performance: a conceptual and empirical approach in higher education. Innov. Educ. Teach. Int. 62, 1–18. doi: 10.1080/14703297.2025.2498529

[ref23] Hodzic S. Scharfen J. Ripoll P. Holling H. Zenasni F. (2018). How efficient are emotional intelligence trainings: a meta-analysis. Emot. Rev. 10, 138–148. doi: 10.1177/1754073917708613

[ref24] Joseph D. L. Newman D. A. (2010). Emotional intelligence: an integrative meta-analysis and cascading model. J. Appl. Psychol. 95, 54–78. doi: 10.1037/a0017286, 20085406

[ref25] Jünger S. Payne S. A. Brine J. Radbruch L. Brearley S. G. (2017). Guidance on conducting and REporting DElphi studies (CREDES) in palliative care: recommendations based on a methodological systematic review. Palliat. Med. 31, 684–706. doi: 10.1177/0269216317690685, 28190381

[ref26] Kolb D. A. Boyatzis R. E. (1970). Goal-setting and self-directed behavior change. Hum. Relat. 23, 439–457.

[ref27] Kotsou I. Mikolajczak M. Heeren A. Grégoire J. Leys C. (2019). Improving emotional intelligence: a systematic review of existing work and future challenges. Emot. Rev. 11, 151–165. doi: 10.1177/1754073917735902

[ref28] Llamas-Díaz D. Cabello R. Megías-Robles A. Fernández-Berrocal P. (2022). Systematic review and meta-analysis: the association between emotional intelligence and subjective well-being in adolescents. J. Adolesc. 94, 925–938. doi: 10.1002/jad.12075, 35860897

[ref9005] MacCann C. Roberts R. D. (2008). New paradigms for assessing emotional intelligence: Theory and data. Emotion 8:540–551. doi: 10.1037/a0012746, 18729584

[ref29] MacCann C. Jiang Y. Brown L. E. R. Double K. S. Bucich M. Minbashian A. (2020). Emotional intelligence predicts academic performance: a meta-analysis. Psychol. Bull. 146, 150–186. doi: 10.1037/bul0000219, 31829667

[ref30] Mattingly V. Kraiger K. (2019). Can emotional intelligence be trained? A meta-analytical investigation. Hum. Resour. Manag. Rev. 29, 140–155. doi: 10.1016/j.hrmr.2018.03.002

[ref31] Mayer J. D. Caruso D. R. (2025). Emotional intelligence and socioemotional attributes: distinct constructs and their uses. Consult. Psychol. J. 77, 219–237. doi: 10.1037/cpb0000293

[ref33] Mayer J. D. Salovey P. (1997). “What is emotional intelligence?” in Emotional Development and Emotional Intelligence: Educational Implications, eds. Salovey P. Sluyter D. J. (New York: Basic Books), 3–34.

[ref32] Mayer J. D. Caruso D. R. Salovey P. (2016). The ability model of emotional intelligence: principles and updates. Emot. Rev. 8, 290–300. doi: 10.1177/1754073916639667

[ref34] Mayer J. D. Salovey P. Caruso D. R. Sitarenios G. (2003). Measuring emotional intelligence with the MSCEIT V2.0. Emotion 3, 97–105. doi: 10.1037/1528-3542.3.1.97, 12899321

[ref35] Naylor R. Baik C. Arkoudis S. (2017). Identifying attrition risk based on the first-year experience. High. Educ. Res. Dev. 37, 328–342. doi: 10.1080/07294360.2017.1370438, 37339054

[ref36] Nelis D. Kotsou I. Quoidbach J. Hansenne M. Weytens F. Dupuis P. . (2011). Increasing emotional competence improves psychological and physical well-being, social relationships, and employability. Emotion 11, 354–366. doi: 10.1037/a0021554, 21500904

[ref37] Niederberger M. Spranger J. (2020). Delphi technique in health sciences: a map. Front. Public Health 8:457. doi: 10.3389/fpubh.2020.00457, 33072683 PMC7536299

[ref38] Palinkas L. A. Horwitz S. M. Green C. A. Wisdom J. P. Duan N. Hoagwood K. (2015). Purposeful sampling for qualitative data collection and analysis in mixed method implementation research. Admin. Pol. Ment. Health 42, 533–544. doi: 10.1007/s10488-013-0528-y, 24193818 PMC4012002

[ref39] Proctor E. K. Bunger A. C. Lengnick-Hall R. Gerke D. R. Martin J. K. Phillips R. J. . (2023). Ten years of implementation outcomes research: a scoping review. Implement. Sci. 18:31. doi: 10.1186/s13012-023-01286-z, 37491242 PMC10367273

[ref9006] Rosenberg M. (1965). Rosenberg Self-Esteem Scale (RSES) [Database record]. APA PsycTests. doi: 10.1037/t01038-000

[ref40] Ruiz-Aranda D. Cabello R. Salguero-Noguera J. M. (2013). Programa Intemo. Guía para mejorar la inteligencia emocional de os adolescentes. Madrid: Ediciones Pirámide.

[ref41] Ruiz-Aranda D. Salguero M. Cabello R. Palomera R. Fernández-Berrocal P. (2012). Can an emotional intelligence program improve adolescents' psychosocial adjustment? Results from the INTEMO project. Soc. Behav. Personal. 40, 1373–1380. doi: 10.2224/sbp.2012.40.8.1373

[ref42] Ruiz-Ortega A. M. Sánchez-Álvarez N. Berrios-Martos M. P. (2024). Psychological well-being and emotional intelligence in undergraduate nursing students as predictors of academic success. Nurse Educ. Today 143:106406. doi: 10.1016/j.nedt.2024.106406, 39288607

[ref9007] Ryan R. M. Connell J. P. (1989). Perceived locus of causality and internalization: Examining reasons for acting in two domains. J. Pers. Soc. Psychol. 57:749–761. doi: 10.1037/0022-3514.57.5.749, 2810024

[ref43] Salovey P. Mayer J. D. (1990). Emotional intelligence. Imagin. Cogn. Pers. 9, 185–211.

[ref44] Salovey P. Mayer J. D. Goldman S. L. Turvey C. Palfai T. P. (1995). Trait Meta-Mood Scale (TMMS) [Database record]. APA PsycTests. doi: 10.1037/t01392-000

[ref45] Sánchez-Álvarez N. Extremera N. Fernández-Berrocal P. (2015). The relation between emotional intelligence and subjective well-being: a meta-analytic investigation. J. Posit. Psychol. 11, 276–285. doi: 10.1080/17439760.2015.1058968, 37339054

[ref46] Santos O. Lopes E. Virgolino A. Stefanovska-Petkovska M. Dinis A. Ambrósio S. . (2018). Defining a brief intervention for the promotion of psychological well-being among unemployed individuals through expert consensus. Front. Psychol. 9:13. doi: 10.3389/fpsyt.2018.00013, 29467680 PMC5808332

[ref47] Schifano J. Niederberger M. (2025). How Delphi studies in the health sciences find consensus: a scoping review. Syst. Rev. 14:14. doi: 10.1186/s13643-024-02738-3, 39810238 PMC11734368

[ref48] Schoeps K. de la Barrera U. Montoya-Castilla I. (2020). Impact of emotional development intervention program on subjective well-being of university students. High. Educ. 79, 711–729. doi: 10.1007/s10734-019-00433-0

[ref49] Sheldon E. Simmonds-Buckley M. Bone C. Mascarenhas T. Chan N. Wincott M. . (2021). Prevalence and risk factors for mental health problems in university undergraduate students: a systematic review with meta-analysis. J. Affect. Disord. 287, 282–292. doi: 10.1016/j.jad.2021.03.054, 33812241

[ref50] Škrinjar I. Vučić K. Puljak L. (2026). Sample size and consensus methods used in Delphi studies are heterogeneous: Meta-research study. J. Clin. Epidemiol. 195:112262. doi: 10.1016/j.jclinepi.2026.112262, 41921829

[ref51] Snape D. Kirkham J. Preston J. Popay J. Britten N. Collins M. . (2014). Exploring areas of consensus and conflict around values underpinning public involvement in health and social care research: a modified Delphi study. BMJ Open 4:e004217. doi: 10.1136/bmjopen-2013-004217, 24413356 PMC3902382

[ref52] Thompson M. (2009). Considering the implication of variations within Delphi research. Fam. Pract. 26, 420–424. doi: 10.1093/fampra/cmp051, 19684122

[ref53] Tinto V. (2017). Through the eyes of students. J. Coll. Stud. Retent. Res. Theory Pract. 19, 254–269. doi: 10.1177/1521025115621917

[ref54] Tuero E. Bernardo A. B. Blanco E. Vázquez-Merino D. (2024). University dropout and transfer intention. What is the influence of resilience and social adaptation on the two types of dropout? J. Coll. Stud. Retent., 28, 1–17. doi: 10.1177/15210251241268812

[ref9008] Watson D. Clark L. A. Tellegen A. (1988). Development and validation of brief measures of positive and negative affect: The PANAS scales. J. Pers. Soc. Psychol. 54:1063–1070. doi: 10.1037/0022-3514.54.6.1063, 3397865

[ref55] Whitsed C. Cassol C. C. Leask B. Morosini M. Elsner C. Nguyen D. (2024). Disrupting internationalisation of the curriculum in Latin America. High. Educ. 88, 1145–1163. doi: 10.1007/s10734-023-01163-0

[ref56] Wong Z. Y. Liem G. A. D. (2022). Student engagement: current state of the construct, conceptual refinement, and future research directions. Educ. Psychol. Rev. 34, 107–138. doi: 10.1007/s10648-021-09628-3

[ref57] Wu Q. Gong H. (2025). Falling into the adaptation “trap”: a follow-up study of first-year students’ adaptation to college. High. Educ. 91, 1063–1086. doi: 10.1007/s10734-025-01456-6

[ref58] Zanden P. Denessen E. Cillessen A. H. Meijer P. C. (2018). Domains and predictors of first-year student success: a systematic review. Educ. Res. Rev. 23, 57–77. doi: 10.1016/j.edurev.2018.01.001

[ref59] Zhoc K. C. H. King R. B. Chung T. S. H. Chen J. (2020). Emotionally intelligent students are more engaged and successful: examining the role of emotional intelligence in higher education. Eur. J. Psychol. Educ. 35, 839–863. doi: 10.1007/s10212-019-00458-0

