# Supplementary material for: Consensus-based guidelines for emotional intelligence interventions for first-year higher education students: a Delphi study
Source: Front Psychol. 2026 Oct 6;17:1866661. doi: 10.3389/fpsyg.2026.1866661 (PMC13680453; doi:10.3389/fpsyg.2026.1866661)
Supplement: Supplementary file 1 [file supplementary_file_1.docx]

Supplementary Material A

# 1 Invitations and Information Sheet for Experts

## 1.1 Round 1

Dear Professor,

We hope to find you well. You are being formally contacted to participate in the international Delphi study focused on developing an emotional intelligence intervention for first-year higher education students. This study is part of the doctoral research project of Fátima Teixeira, funded by the Portuguese Foundation for Science and Technology (2023.04864.BD) and supervised by Alexandra Araújo, PhD (Universidade Portucalense, Portugal) and Rosario Cabello, PhD (Universidad de Málaga, Spain).

Your contribution will be key to this study and will be most appreciated. This Delphi study will occur between June and July 2024. It will gather a group of experts to complete a survey with questions about the core components of this intervention, while also focusing on students’ adaptation to higher education, well-being, and academic success.

The Delphi survey is based on international guidelines to report group-based interventions and includes three sections: design, content, and assessment plan.

More information about the study is attached to this e-mail in the document “**Delphi Study_Information for panelists**”.

Your participation is voluntary, and we will guarantee confidentiality and anonymity under ethical guidelines for research in psychology.

Before answering the survey, please accept the conditions and ethical requirements to participate in this study and answer a few questions about your background expertise. After this, the Delphi Survey will start. We ask you to submit your answers by the end of June 30th.

Please, enter this link to accept the conditions and start the survey:

Any questions, please get in touch with the team by e-mail.

## 1.2 Round 2

Dear Expert,

Thank you for participating in the Delphi Round 1 Survey!

Your responses to the first questionnaire were thorough and relevant. We received a piece of much-appreciated advice and guidance, which is well supported by your personal and professional experience. We have now analyzed your answers and comments and you can access a summary of the results here: “Link”

Overall, consensus was reached for most questions, and for the ones we intended to select the better options (e.g., session activities), we were able to select a set of recommendations. Nonetheless, we couldn´t reach a consensus on 2% of the questions. We ultimately want to reach a general agreement, so the purpose of Round 2 is to steer the group toward consensus.

Therefore, when designing Delphi Survey Rond 2 we included questions that didn´t reach consensus in Round 1, by rephrasing them, changing the response scale, excluding options with low acceptance, or including options suggested by the panel. We have tried to include all the suggestions wherever we had to choose the ones that fit the most in our study, for different reasons (e.g., not including measures not validated in undergraduate students or to the Portuguese population).

Delphi Round 2

The Delphi Round 2 Survey should take around 10 minutes to complete. It is structured differently from the first questionnaire because we grouped the statements to avoid a long questionnaire. Nonetheless, it follows the same sequence as questions in the 1st Round. A commentary box will be available at the end if you want to comment, justify, or give feedback.

The survey contains a list of statements that we need you to agree/decline or select between two options. We will present you with the results of these answers after this round.

The closing date for your responses to Round 2 is 31st of July 2024. Please enter this link to answer Round 2: “link”

**End of Study**

Dear Expert,

Thank you for contributing and taking the time to complete our Delphi survey!

Your participation is immensely appreciated and was incredibly valuable to our study. Your perspective and insights were key to the design of our intervention.

The results of the second round will be available and shared in September.

Until then, our team wishes you a well-deserved break and a wonderful vacation.

# 2 Delphi Study: Information for panelists

Objective of the study:

This study aims to identify the components of an Emotional Intelligence group-based intervention to promote adaptation to higher education (HE), academic success, and mental health in first-year HE students, by conducting a Delphi study with international experts on the promotion of socioemotional competencies.

The survey

The Delphi survey is based on international guidelines to report group-based interventions and includes three sections: design, content, and assessment plan of a group-based EI intervention.

**Section 1** - Intervention design (number of group sessions, length of sessions, frequency, duration of the interventions, participants' material, activities during and after sessions, types of activities, group sizes, and facilitators).

**Section 2** - Intervention content (the content of the sessions, and sequencing of sessions).

**Section 3** - Efficacy assessment (assessment plan, time points, and assessment tools).

Answers

The survey has two answer formats: statements that must be answered on a Likert scale and multiple-choice answers. All que questions have an open box for comments or additional information. The survey has 22 main questions.

Rounds

For this study, **two rounds** are planned with an extra round if disagreement lingers.

**Round 1** - Sociodemographic information about panelists, Delphi survey (LimeSurvey link), optional online meetings for questions. Deadline: 2 weeks; Feedback (1 week after).

**Round 2** - Delphi survey with questions that didn’t reach an agreement (Lime Survey link), optional online meetings for questions. Deadline: 1 week; Feedback (1 week after).

**Round 3** - Same as Round 2.

**The Delphi study's final results and the intervention's pilot protocol will be first-hand presented to the panel in an online seminar by the end of the study.**

If you have any questions, don't hesitate to get in touch with the research team using the following e-mail addresses:

- Fátima Teixeira – [fatima.teixeira@upt.pt](mailto:fatima.teixeira@upt.pt) |Alexandra M. Araújo - [amaraujo@upt.pt](mailto:amaraujo@upt.pt) | Rosario Cabello - [rcabello@uma.es](mailto:rcabello@uma.es)

**Thank you for collaborating on this study!**

# 3 Informed Consent (via LimeSurvey)

**Ethical Requirements**

Participants of the Delphi study must contribute based on his/her own opinions or the opinions of the organization they are representing. The study will be conducted entirely online and it is requested that the participants avoid discussing their opinions with other members. Online meetings with the moderator (Fátima Teixeira) can be scheduled to clarify questions.

Anonymity of the participant's and individual's responses would not be used in any way that would allow his/her identification. However, permission is requested to list participants' names, and names of their organizations, as part of the Delphi panel after the Delphi study is completed.

The participants can withdraw from the project at any time.

Select all that apply:

___I agree to participate in the project

___I will not discuss my opinions and answers with other members of the panel

___I agree to disclose my name and the name of my organization at the end of the project for publishing purposes.

- The Responses and IP address were anonymized using LimeSurvey settings.
