# Supplementary material for: Consensus-based guidelines for emotional intelligence interventions for first-year higher education students: a Delphi study
Source: Front Psychol. 2026 Oct 6;17:1866661. doi: 10.3389/fpsyg.2026.1866661 (PMC13680453; doi:10.3389/fpsyg.2026.1866661)
Supplement: Supplementary file 2 [file supplementary_file_2.docx]

Supplementary Material B

# 1 Supplementary Tables

| **Table B1**  *Sociodemographic Information of The Delphi Expert Panel* | | | |
| --- | --- | --- | --- |
|  |  | ***n*** | **%** |
| **Country** | Portugal | 4 | 33.3 |
|  | Spain | 3 | 25 |
|  | Italy | 2 | 16.7 |
|  | Brazil | 1 | 8.3 |
|  | Chile | 2 | 16.7 |
| **Field of expertise** | Implementation of psychological interventions (i.e., educational or clinical) | 9 | 75 |
|  | Promotion of emotional intelligence | 10 | 83.3 |
|  | Promotion of socioemotional competences | 9 | 75 |
|  | Intervention in educational settings | 7 | 58.3 |
|  | Intervention in higher education | 5 | 41.7 |
|  | Promoting academic success | 5 | 41.7 |
|  | Promoting mental health | 10 | 83.3 |
| **Professional discipline** | Clinical psychology | 6 | 50 |
|  | Educational psychology | 8 | 66.7 |
| **Professional role** | Researcher | 8 | 66.7 |
|  | Professor | 9 | 75 |
|  | Psychologist (i.e., clinical practice) | 7 | 58.3 |
| **Highest qualification** | Master´s degree | 2 | 16.7 |
|  | PhD | 6 | 50 |
|  | Postdoc | 4 | 33.3 |
| **Experience in the expertise field** | < 2 years | 1 | 8.3 |
|  | 5 – 10 years | 3 | 25 |
|  | > 10 years | 8 | 66.7 |
| **Publications in the expertise field** | < 2 publications | 1 | 8.3 |
|  | 5-10 publications | 3 | 25 |
|  | >10 publications | 8 | 66.7 |

| **Table B2**  *Items with Non Consensual Results Submitted to Experts in Round 1 and 2* | | | | | | | |
| --- | --- | --- | --- | --- | --- | --- | --- |
| **Dimensions** | **Subdimensions** | **Options** | ***Round*** |  | ***n*** | **% agreement** |  |
| **Intervention design** | **Total number of group sessions** | 6 sessions  7sessions  other | 1^st^ |  | 3  3  6 | 25  25  50 |  |
|  |  | A total of 8 sessions: two per emotional ability (perception, use, understanding, management) with the introductory session, and a final session included | 2^nd^ |  | 3 | 25 |  |
|  | **Missing sessions** | 0 % (i.e., 0 sessions)  10%  20%  Other | 1^st^ |  | 3  4  5  0 | 25  33.3  41.7  0 |  |
|  |  | Can skip 2 sessions, if not consecutive (i.e., about the same emotional ability) | 2^nd^ |  | 5 | 25 |  |
|  | **Group composition** | Between 6 and 8 participants  Between 8 and 15 participants  Between 15 and 20 participants | 1^st^ |  | 3  5  4 | 25  41.7  33.3 |  |
|  |  | More than 15 participants (large group) | 2^nd^ |  | 1 | 8.3 |  |
|  | **Group composition criteria*** | Students’ schedules for the semester  Students’ BSc or scientific field  Age (e.g., 1st year students that are older than the average age of 18 years) | 1^st^ |  | 7  4  6 | 58.3  33.3  50 |  |
|  |  | Sociodemographic characteristics  Age (i.e., Different groups for younger and older students)  Scientific field  Mental health risk levels | 2^nd^ |  | 1  5  2  6 | 8.3  41.7  16  50 |  |
|  | **Type of activities*** | More individual activities than group activities  More group activities than individual activities  The same amount of individual and group activities | 1^st^ |  | 2  6  4 | 16.7  50  33.3 |  |
|  | **Session activities*** | E-learning tutorials  Reflection papers  Cards  Metaphors  Storytelling  Dilemma discussions | 1^st^ |  | 5  3  2  3  6  2 | 41.7  25  16.7  25  50  16.7 |  |
|  | **Partcicipant’s materials*** | A list of additional support resources  Online tutorials | 1^st^ |  | 6  5 | 50  41.7 |  |
|  | **Activities between sessions (i.e., homework)*** | Reflexive journals  Participation in a group chat  Other | 1^st^ |  | 5  6  2 | 41.7  50  16.7 |  |
| **Intervention content** | **Additional competencies*** | Academic success competencies  No | 1^st^ |  | 6  3 | 50  25 |  |
|  | **Personal competencies*** | Autonomy and interdependence  Vocational clarity  Other | 1^st^ |  | 5  2  1 | 41.7  16.7  8.3 |  |
|  | **Mental health and well-being*** | Physical health and daily routines  Risk behaviors  Other | 1^st^ |  | 6  5  1 | 50  41.7  8.3 |  |
|  | **Academic success competencies*** | Relationship with teachers  Involvement in extracurricular activities on campus  Other | 1^st^ |  | 6  2  0 | 50  16.7  0 |  |
|  | **Sequence of the sessions** | Separate contents (i.e., sessions focused on Emotional Intelligence abilities and sessions focused on other competencies, separately) | 1^st^ |  | 1 | 8.3 |  |
| **Intervention efficacy assessment** | **Methods for efficacy assessment** | Quantitative  Qualitative | 1^st^ |  | 0  0 | 0  0 |  |
|  | **Quantitative assessment*** | Peer-report  Other | 1^st^ |  | 6  0 | 50  0 |  |
|  | **Qualitative assessment*** | Case study  Other | 1^st^ |  | 1  1 | 8.3  8.3 |  |
|  | **Emotional Intelligence assessment tools*** | Vocabulary of Emotions Test (VET; Costa et al., 2011)  Wong and Law Emotional Intelligence Scale (WLEIS; Wong & Law, 2002)  Situational Test of Emotion Management (STEM; Palmer & Stough, 2001)  Situational Test of Emotion Understanding (STEU; Palmer & Stough, 2001)  Other | 1^st^ |  | 6  3  6  5 | 50  25  50  41.7 |  |
|  | **Additional assessment measures*** | Other | 1^st^ |  | 2 | 16.7 |  |
|  | **Academic Success Assessment*** | Utrecht Work Engagement Scale-Students | 1^st^ |  | 5 | 41.7 |  |
|  | **Control variables*** | Use of psychopharmaceuticals  Other | 1^st^ |  | 6  3 | 50  25 |  |
|  | **Pre-test assessment**  **Post-test assessment** | 1st session | 1^st^ |  | 2 | 16.7 |  |
|  |  | Last session  1 week after the intervention  2 weeks after the intervention  Other | 1^st^ |  | 4  5  2 | 33.3  41.7  16.7 |  |
|  |  | In the last session after addressing all the content |  |  | 6 | 50 |  |
|  |  | In a separate session after the intervention | 2^nd^ |  | 6 | 50 |  |
|  | **Number of follow-up moments** | 0 moments  2 moments | 1^st^ |  | 3  1 | 25  8.3 |  |
|  | **Session assessment*** | Other | 1^st^ |  | 3 | 25 |  |
| **Dimensions** | **Subdimensions** | **Statements for agreement or importance raking** | **Round** | ***M*** | ***Coefficient of variation*** | **% of agreement**** |  |
| Intervention design | ªGroup composition | Include criteria in the composition of groups | 1^st^ | 342 | 1.17 | 58.4 |  |
| Intervention efficacy assessment | Session Assessment | Include the assessment of sessions | 1^st^ | 3 | 1.34 | 41.6 |  |
| *Note.* “Round” indicates in which round the item was resolved; *Yes/ No response for including each option; *”4” and “5” values on a 5-point Likert scale, ^a^item included only in Round 2. | | | | | | |  |
|  |  |  |  |  |  |  |  |

| **Table B3**  *Expert Comments and Suggestions for New Questions* | | | |
| --- | --- | --- | --- |
| **Dimensions** | **Subdimensions** | **Experts Statement** | **Generated items/ Output for 2^nd^ Round** |
| **Intervention design** | **Number of sessions** | *8 to dedicated two sessions to each EI ability*  *A total of 10 sessions, two per area (perception, use, understanding, management), an introductory session and a final session.*  *Between 10 and 20 sessions, plus follow-up*  *More than seven sessions*  *10 sessions at least*  *8 sessions at minimum* | 8 sessions  10 sessions |
|  | **Missing sessions** | *The lower percentage the better; perhaps consider below 10%* | Can skip 1 session  Can skip 2 sessions, if not consecutive (i.e., about the same emotional ability) |
|  | **Group composition criteria** | *Students at mental health risk*  *Group homogeneous regarding the year of education* | Age (i.e., different groups for younger and older students)  Mental health risk levels |
|  | **Number of facilitators** | *depending on the number of trainees perhaps 2 facilitators could be beneficial*  *Using 2 facilitators tends to improve adherence to the intervention protocol* | Included as recommendations for future interventions |
|  | **Session activities** | *Assessment and self-assessment of emotional abilities*  *Relaxation exercises* | Monitor emotions, thoughts, and experiences during the intervention  Relaxation exercises |
|  | **Participants’ materials** | *audios for some more experiential/ mindfulness practices*  *app to help them to monitor their emotions during the period of training* | Included as recommendations for future interventions |
|  | **Activities between sessions** | *Diary or something else that helps to monitor emotion and thinking*  *homework activities according to the content of each session*  *Use monitoring apps or wearable devices*  *Worksheets for practices between sessions to apply the competencies learned in the session* | Worksheets for practices between sessions (include on participant's materials) |
| **Intervention content** | **Personal competences** | *incluir temáticas de como el cuidado y bienestar de la salud mental*  *Potenciar la gestión emocional ante procesos de frustración que puedan pasar en su primer año académico* | Included as recommendations for future interventions |
| **Intervention efficacy assessment** | **Additional tools** | *DERS: One subscale assesses the lack of emotional acceptance scale; the test MSCEIT; the IE-ACCME tests; Eudaimonic Well-Being; ASEBA by Achenbach; Pemberton Happiness Index*  *Satisfaction with life scale; Escala de Autocompaixão de Neff;; Depression Anxiety and Stress Scale (DASS-21); Escala de Esperança Cognitiva - Escala de Orientação da Vida Revisado – LOT-R Escala de Autoeficácia Geral; Escala de Funcionamento Psicológico Positivo (EFPP)* | Included as recommendations for future interventions |
|  | **Control variables** | *Trait features*  *Given the high prevalence of anxiety and depression in students, I recommend including one measure of psychopathological symptoms, such as Depression Anxiety and Stress Scales*  *Practicing sports or leisure activities, psychotherapy, use of alcohol or other drugs, psychiatric diagnoses or psychiatric symptoms, school failures quality of family relationships, social support networks* | Psychopathological symptoms, such as depression and anxiety  Trait features (ie., personality traits)  Included as recommendations for future interventions |
