# Supplementary material for: Consensus-based guidelines for emotional intelligence interventions for first-year higher education students: a Delphi study
Source: Front Psychol. 2026 Oct 6;17:1866661. doi: 10.3389/fpsyg.2026.1866661 (PMC13680453; doi:10.3389/fpsyg.2026.1866661)
Supplement: Supplementary file 3 [file supplementary_file_3.docx]

Supplementary Material C

Guidelines for an Emotional Intelligence Intervention for
First-Year Higher Education Students

Fátima M. Teixeira, Alexandra M. Araújo & Rosario Cabello

| Guidelines for an Emotional Intelligence Intervention for  First-Year Higher Education Students | | | |
| --- | --- | --- | --- |
| **Dimensions** | **Subdimensions** | **Elements** | **Included?** |
| **Intervention Design** | **Total number of group sessions** | 10 sessions: two per emotional ability (perception, use, understanding, management), an introductory session, and a final session |  |
|  | **Length of group sessions** | 2h |  |
|  | **Frequency of group sessions** | 1 x week |  |
|  | **Duration of the intervention** | An intervention distributed over 1 semester, preferably in the first semester |  |
|  | **Missing sessions** | Can miss 1 session |  |
|  | **Number of participants per group** | Up to 15 participants (medium group) |  |
|  | **Group composition criteria** | Students’ schedules and availability |  |
|  | **Number of facilitators** | 2 facilitators |  |
|  | **Type of activities** | The same amount of individual and group activities |  |
|  | **Session activities** | Roleplays  Class discussions  Activities feedback  Individual training tasks*  Team assignments  Educational content (i.e., emotional literacy)  Videos  Case studies (presentation and discussion of real-life cases and solutions)*  Association between emotions and life events  Display of emotional vocabulary  Debates*  Games adapted to emotional content  Monitor emotions, thoughts, and experiences during the intervention  Relaxation exercises |  |
|  | **Intervention materials** | General information about the intervention and objectives |  |
|  |  | A support guide about the intervention and individual sessions |  |
|  |  | Fact sheets summarizing each session |  |
|  |  | Support material about session exercises |  |
|  | **Activities between sessions (i.e., homework)** | Specific prescribed activities related to the sessions and worksheets for practice |  |
| **Intervention content** | **EI abilities** | Perceiving, using, understanding, and managing emotions  (With more time allocated to understanding and managing emotions) |  |
|  | **Additional competencies to embed in the contents** | Personal competencies  Mental health and well-being |  |
|  | **Personal competencies** | Identity and self-esteem*  Interpersonal relationships*  Empathy* |  |
|  | **Mental health and well-being themes** | Well-being and self-care |  |
|  |  | Stress management and coping strategies |  |
|  |  | Mental health (i.e., anxiety, stress, and depression) |  |
|  |  | Emotional regulation strategies |  |
|  | **Academic success competencies** | Self-regulated learning* |  |
|  |  | The balance between academic, social, and personal life* |  |
|  |  | Relationship with peers |  |
|  | **Sequence of the sessions** | Focus primarily on the emotional abilities and gradually embed additional competencies related to students’ academic life and well-being |  |
| **Intervention Assessment** | **Methods for efficacy assessment** | Mixed-methods |  |
|  | **Quantitative assessment** | Self-report |  |
|  |  | Ability tests |  |
|  | **Qualitative assessment** | Individual interviews* |  |
|  |  | Focus group |  |
|  | **EI assessment tools suggested** | Trait Meta Mood Scale (TMMS; Fernandez-Berrocal & Extremera, 2004) |  |
|  |  | Situational Test of Emotion Management (STEM; Palmer & Stough, 2001) |  |
|  | **Additional assessment measures suggested** | Positive And Negative Affect Scale (PANAS; Watson et al., 1988) |  |
|  |  | Satisfaction With Life Scale (SWLS; Diener et al., 1985)* |  |
|  |  | Rosenberg Self-Esteem Scale (Rosenberg, 1965) |  |
|  | **Academic Success Assessment suggested** | Academic GRADES |  |
|  |  | Self-Regulation Questionnaire-Academic |  |
|  | **Control variables** | Generalized self-efficacy (GSE; Schwarzer & Jerusalem, 1995) |  |
|  |  | Motivation for the intervention |  |
|  |  | Scientific field* |  |
|  |  | Gender |  |
|  |  | Age |  |
|  |  | Family educational background |  |
|  |  | Mental health issues, such as depression and anxiety |  |
|  |  | Trait features (i.e., personality traits) |  |
|  | **Pre-test assessment** | 1 week before the intervention |  |
|  | **Post-test assessment** | 1 week after the intervention^+^ |  |
|  | **Follow-up moments** | 1 moment |  |
|  | **Session assessment** | Level of satisfaction with the session |  |
|  |  | Level of satisfaction with what was learned |  |
|  |  | Level of perceived utility of the session |  |
|  |  | Commentary section (e.g., suggestions for future sessions) |  |
| *Note.* Recommendations meeting the predefined Delphi consensus threshold constitute the expert consensus-based guidelines presented in this document. Items labelled as near-consensus findings (identified with an asterisk [*]) did not reach the predefined consensus threshold and should therefore not be interpreted as expert consensus-based recommendations. They are included to inform future research and intervention development and may be considered, with caution, according to the specific objectives and context of the intended intervention.  **^+^Author-informed suggestion:** As no formal Delphi consensus was reached regarding the timing of the post-intervention assessment, a follow-up approximately one week after program completion is proposed based on the rationale presented in the Discussion and should be further examined in future research. | | | |
